# Subjective and Functional Health Literacy Screening in Geriatric Primary Care

**DOI:** 10.1093/geroni/igaa057.1221

**Published:** 2020-12-16

**Authors:** Amy Albright, Deanna Dragan, Anne Halli-Tierney, Dana Carroll, Rebecca Allen

**Affiliations:** 1 University of Alabama, Northport, Alabama, United States; 2 The University of Alabama, Tuscaloosa, Alabama, United States; 3 Auburn University Harrison School of Pharmacy, Tuscaloosa, Alabama, United States; 4 Alabama Research Institute on Aging, Tuscaloosa, Alabama, United States

## Abstract

The aim of the current study is to provide comprehensive health care to older adults by assessing physical and mental health in a geriatric primary care setting, including evaluation of both subjective and functional health literacy. Health literacy is vital to understanding medical information and making subsequent decisions based on this information. Knowledge of patient health literacy may be particularly important for care providers, as it can provide guidance on how to best communicate with the patient (Nouri & Rudd, 2015). It may be particularly important to monitor health literacy within older adults, as several studies (e.g., Kobayashi et al., 2015) have shown that health literacy decreases with mild cognitive impairment. Approximately 250 patients (mean age = 76; 74% female; 16% African American) attending an interdisciplinary geriatrics clinic in West Alabama have been recruited to take part in a variety of behavioral health screenings. The current study assessed subjective health literacy using questions developed by Chew, Bradley, and Boyko (2004) and functional health literacy using the Newest Vital Sign (Weiss et al., 2005). While there was a significant correlation between subjective and functional health literacy (r = .43, p < .001), 81% of patients reported adequate subjective health literacy, while only 41% demonstrated adequate health literacy on a functional screening measure. Based on these findings, self-reported health literacy may not necessarily be reflective of performance on more functional measures. Given the potential consequences of overestimating health literacy, this represents a serious barrier to patient care.

